# The Circulation of the Blood

**Published:** 1890-09

**Authors:** 


### SELECTIONS.

THE CIRCULATION OF THE BLOOD.—When the blood, driven by the all powerful heart of man, rolls its crimson tide through the thousand canals that permeate the body, it glorifies life, giving vigor to the feet and splendor to the eyes; while the brain, hidden under a vaulted dome, is filled with beautiful thoughts and brilliant ideas. When the blood feels its sovereign heat failing, it re-enters the lung, dark, feeble, and unhealthy; but the pure air of heaven, with its revivifying health, touches with strength and renews life's spirit with its divine element. Rich with nourishment, the blood again descends the human edifice, red and pure as old wine. 'Tis thus the soul supports the body in the universe; 'tis thus the soul traverses the earth and stars and visits the innumerable constellations; 'tis thus the soul courses through space unfathonable, burning in the air in sublime flames, glittering in masses of gold 'mid forms of flowers and animals, communicating to nature all its strength and grace. When the soul, losing its heated blood, feels its warmth at last fading away with the enfeeblement of the fiery fluid, it returns to its primitive source of supreme strength and seeks once more the air, the breath of God.—*August Rarbier.*

